# Cross-modal functional plasticity after cochlear implantation

**DOI:** 10.1093/cercor/bhaf084

**Published:** 2025-04-22

**Authors:** Jamal Esmaelpoor, Tommy Peng, Beth Jelfs, Darren Mao, Maureen J Shader, Colette M McKay

**Affiliations:** Department of Medical Bionics, University of Melbourne, 29 Royal Parade, Parkville, VIC 3052, Australia; Bionics Institute, 41 Victoria Parade, Fitzroy, VIC 3065, Australia; Department of Medical Bionics, University of Melbourne, 29 Royal Parade, Parkville, VIC 3052, Australia; Bionics Institute, 41 Victoria Parade, Fitzroy, VIC 3065, Australia; Department of Electronic, Electrical and Systems Engineering, University of Birmingham, Edgbaston, Birmingham, B15 2TT, United Kingdom; Department of Medical Bionics, University of Melbourne, 29 Royal Parade, Parkville, VIC 3052, Australia; Bionics Institute, 41 Victoria Parade, Fitzroy, VIC 3065, Australia; Department of Speech, Language, and Hearing Sciences, Purdue University, 715 Clinic Drive, West Lafayette, IN 47907, United States; Department of Medical Bionics, University of Melbourne, 29 Royal Parade, Parkville, VIC 3052, Australia; Bionics Institute, 41 Victoria Parade, Fitzroy, VIC 3065, Australia

**Keywords:** cross-modal plasticity, cochlear implants, functional connectivity, fNIRSoutcome prediction, psychophysiological interactions

## Abstract

Despite evidence that cross-modal effects after hearing loss and cochlear implantation are primarily driven by synaptic gain and efficacy, few studies have evaluated cross-modal functional connectivity (CMFC) to assess plasticity. This study, inspired by the psychophysiological interaction (PPI) method, addresses its limitations and provides a robust approach for assessing task-induced CMFC. Twenty-three postlingually deafened cochlear implant (CI) recipients and 17 normal-hearing (NH) participants took part in the study. Functional near-infrared spectroscopy was used to measure brain activity during audio-only and visual-only speech tasks, with resting-state FC as a baseline, at 1 month and 1 year postimplantation. CI users’ speech understanding was assessed 1 year postimplantation. Significant negative correlations were observed between contralateral task-induced CMFC and speech outcomes, particularly in links from the angular gyrus (AG) to the visual cortex. One year after CI activation, higher task-induced CMFC was found in AG compared to the superior temporal gyrus, reflecting neural efficiency principles. Task-induced CMFC remained elevated in CI users compared to NH even after 1 year. These findings suggest task-induced CMFC as a significant marker of cross-modal plasticity and speech performance in CI recipients.

## Introduction

Loss of a sensory modality, such as hearing in cases of postlingual hearing loss, causes a phenomenon known as cross-modal plasticity in the brain. Cross-modal plasticity is characterized by the need to compensate for the deprived sensory modality resulting in heightened brain activity in regions associated with the remaining senses, and the primary cortical area previously dedicated to the lost modality undergoes a takeover by other senses (Bavelier and Neville 2002; Merabet and Pascual-Leone 2010). Notably, individuals with severe hearing loss often adapt by relying more on intact senses such as vision. In these cases, the auditory cortex becomes increasingly responsive to visual stimuli, enhancing capabilities in visual object localization and motion detection (Buckley and Tobey 2011; Mao and Pallas 2013; Kral and Sharma 2023).

Cochlear implants offer a partial restoration of hearing ability, for individuals with severe hearing loss, through electrical stimulation of ganglion cells in the auditory nerves. The introduction of auditory stimulation postcochlear implantation (CI) triggers cross-modal plasticity in visual and auditory cortices, enabling the brain to adapt to novel stimuli. However, post-CI plasticity is complex and multifaceted. First, sensorineural hearing loss precipitates various changes, including a significant reduction in spiral ganglion neurons, demyelination of residual neurons, shrinkage of perikaryon in the auditory pathway, and a decrease in spontaneous activity in the auditory pathway (Fallon et al. 2008). Second, the implant delivers signals with coarse spectral content compared to natural acoustic hearing (Wilson and Dorman 2008). Consequently, postlingual implant recipients must forge new associations with the sounds received after implantation, often necessitating additional neural resources, such as increased cognitive load and continued reliance on supplementary cues like lipreading (Giraud et al. 2001; Fullerton et al. 2023).

Initial animal model studies in the field have demonstrated that when deafness occurs early in life and is combined with the removal of the auditory midbrain, it results in the reorganization of anatomical inputs to the thalamus and a large-scale remapping of the auditory cortex by visual inputs (Sur et al. 1990; Von Melchner et al. 2000). Influenced by this perspective, many studies suggested that the entire auditory cortex becomes a battleground for sensory systems, with each area potentially being recruited for a new sensory function in the absence of auditory input (Irvine and Huebner 1979; Bowman and Olson 1988). However, recent findings challenge this notion, refuting the idea that massive cross-modal reorganization occurs in the auditory system after hearing loss without anatomical rerouting of visual information. These findings suggest that the cause of cross-modal reorganization is primarily synaptic and functional, confined to restricted auditory areas. The brain can repurpose the same neuronal circuitry for qualitatively different functions (Kral and Sharma 2023). Cross-modal effects are conveyed predominantly through synapses, their number, and synaptic efficacy rather than reorganized fiber tracts. Therefore, assessing functional connectivity (FC), which involves evaluating statistical dependencies between activities in different brain areas (Fornito et al. 2016), appears to be a reasonable approach for understanding changes related to cross-modal reorganization.

Even though cross-modal regional activation has been widely assessed by many studies (Giraud et al. 2001; Lee et al. 2001; Buckley and Tobey 2011; Mao and Pallas 2013; Strelnikov et al. 2013; Campbell and Sharma 2016), there are only a limited number of studies that have explored cross-modal functional connectivity (CMFC) for postcochlear implantation users (Chen et al. 2017; Fullerton et al. 2023). These studies on CMFC exhibit disparities in their conceptualizations of FC and the specific aspects they evaluate. For example, Chen et al. investigated task-induced CMFC between the visual and auditory cortices during both visual and auditory tasks among CI users and individuals with normal hearing (NH) (Chen et al. 2017). Employing functional near-infrared spectroscopy (fNIRS) for brain imaging, they selectively excluded channels demonstrating the highest activation during visual and auditory tasks in their respective areas. This selection criterion stemmed from the presumption that cross-modal changes predominantly occur in secondary areas. Additionally, they normalized CMFC, measured via Pearson correlation, by subtracting average intramodal connectivity between channels in the left and right hemispheres, assuming negligible task effects on intramodal connectivity and regional disparities in baseline FC. The outcomes revealed heightened task-induced CMFC in CI users compared to the normal hearing group. Moreover, the discrepancy in CMFC for visual and auditory stimuli correlated with speech recognition outcomes in CI users.

In contrast, Fullerton et al. focused on channels exhibiting the highest task activation for CMFC analyses, also using fNIRS for brain imaging (Fullerton et al. 2023). They quantified FC between channels using coherence, focusing on the raw values of FC between channels. This approach potentially assumes minimal residual physiological noise after band-pass filtering, or negligible individual/regional differences in physiological noise, which, given the characteristics of fNIRS signals, would be strong assumptions (Santosa et al. 2020). Their findings highlighted a positive correlation between connectivity in the left auditory and visual cortices and CI users’ abilities to comprehend speech in background noise. However, they overlooked the transient effects of tasks when evaluating FC during the task. These divergent methodologies underscore the complexities and variations inherent in investigating cross-modal FC within the realm of auditory rehabilitation.

An alternative method for investigating task-specific changes in FC is psychophysiological interaction (PPI) analysis, introduced by Friston et al. (Friston et al. 1997). PPI assesses changes in the relationship between activity in different brain areas using a regression model with three regressors for each seed channel. The main regressor of interest is the psychophysiological interaction; however, to obtain this, we must first consider the interactions that are either purely psychological or purely physiological. To rule out the transient effect of the task itself (psychological interaction), a regressor is obtained by convolving a boxcar function representing the duration of the task with the hemodynamic response function (HRF) to give the HRF-convolved task. To account for the anatomical connections with the seed channel (physiological interaction), the time course from the seed channel of interest is used as a regressor. This accounts for the correlations present all the time, both during and outside of the task duration. The final regressor is the element-wise product of the HRF-convolved task and seed channel. This covariate represents the psychophysiological interaction and accounts for the changes in FC due to the task (O’Reilly et al. 2012). The generalized form of PPI for scenarios including two tasks examines the difference in effects of different tasks on baseline FC and does not provide their effects separately (McLaren et al. 2012).

This study investigates task-induced CMFC in postlingually deaf CI users, focusing on the interaction between visual and auditory brain regions during visual and audio tasks and its correlation with speech comprehension 1 year after cochlear implantation. fNIRS, a noninvasive imaging technique that measures cortical brain activity by detecting changes in blood oxygenation and blood volume without interfering with the CI device (Shin et al. 2024), was used for recordings at two time points: 1 month and 1 year postimplantation. To achieve these aims, we devised an approach inspired by PPI. However, we acknowledge that utilizing PPI itself imposes certain limitations. To address these constraints, we opted for resting-state recordings instead of short intervals between task trials (control trials) to ascertain baseline FC and to deal with the restriction of generalized PPI for scenarios including two tasks (McLaren et al. 2012). Our primary hypothesis is that task-induced CMFC correlates with the behavioral speech outcomes of CI users. Our primary hypothesis is that task-induced CMFC correlates with the speech outcomes of CI users. We initially evaluate this correlation across broad auditory areas covered by the montage. Building on these findings, we take exploratory steps to analyze restricted regions associated with primary and secondary auditory areas, addressing discrepancies in previous studies regarding cross-modal FC. Lastly, we compare CMFC in CI recipients with that of an NH group to identify potential disparities.

## Materials and methods

### Participants

We enrolled 26 postlingually deafened newly implanted adult CI recipients with severe hearing loss in the contralateral ear (pure-tone average (PTA) of 0.5, 1, 2, and 4 kHz > 45 dBHL, where dBHL stands for decibels Hearing Level) for this study. As the study evaluates task-induced changes in FC with resting-state serving as the baseline, three subjects were excluded due to missing resting-state recordings. Consequently, data from 23 CI recipients were utilized (mean age = 58 ± 14 years, 14 male, 9 female; demographic information in Table 1). Additionally, 17 age-matched NH subjects with PTA < 45 dBHL in both ears participated in the experiment (mean age = 58 ± 14 years, 8 male, 9 female). Ethics approval for this study was obtained from the Royal Victorian Eye and Ear Hospital (ethics approval 19.1418H), and all participants provided informed written consent.

**Table 1:** Demographic information of participants.

Participant gender		Age (years)	Implant ear	Deafness duration (years)	Nonimplant ear PTA^a^ (dBHL)
Sub-01	M	62	R	N.R.*^b^*	64
Sub-02	F	48	R	20	56
Sub-03	F	56	R	48	76
Sub-04	M	74	R	60	92
Sub-05	F	75	R	30	111
Sub-06	M	61	R	30	66
Sub-07	M	30	L	10	106
Sub-08	M	65	L	27	72
Sub-09	M	62	L	15	74
Sub-10	M	43	R	30	69
Sub-11	F	43	R	0.17	87
Sub-12	M	33	L	7	101
Sub-13	M	58	R	45	65
Sub-14	M	68	R	43	120
Sub-15	F	67	L	N.R.	97
Sub-16	F	68	R	28	76
Sub-17	F	61	R	25	81
Sub-18	M	71	R	30	104
Sub-19	M	34	R	25	71
Sub-20	F	62	L	30	96
Sub-21	M	75	R	20	80
Sub-22	F	71	R	8	67
Sub-23	M	54	R	N.R.	71

N.R., no record.

^a^PTA (dBHL) = pure-tone average of 0.5 1 and 2 4 kHz hearing thresh- olds.

Given the older age group of our participants, their cognitive skills were assessed to identify any potential cognitive decline. Cognitive skills were evaluated using trial-making tests A and B (Ashendorf et al. 2008). Test A required participants to draw lines between numbers in ascending order, while Test B involved drawing lines alternately between numbers and letters in ascending order. Both tests were performed on a sheet of paper with random placements. Participants were instructed to complete the tasks quickly and accurately without lifting the pencil. All participants demonstrated normal performance, meeting the specified criteria (Tombaugh 2004).

CI outcomes were assessed 1 year after device activation. Participants underwent two audio-only speech tests to measure speech understanding performance. The first test involved 50 consonant–nucleus–consonant (CNC) words presented in quiet (Peterson and Lehiste 1962). The second test comprised 15 Bamford–Kowal–Bench (BKB) sentences presented in four-talker babble noise (at a signal-to-noise ratio [SNR] = 10 dB) (Dawson et al. 2013). Participants were seated comfortably and experienced free-field presentation of speech stimuli at 65 dBA through their implants. To isolate the participant’s performance with the implant, the contralateral ear was occluded and masked when appropriate. Test scores for the first test were based on the percentage of correct phonemes recognized, while, for the second test, it was the percentage of correct words in repeated sentences. The speech score results were transformed into rationalized arcsine units for statistical analysis (Sherbecoe and Studebaker 2004).

### Experiments

We conducted a procedure comprising a task-based experiment with audio and visual tasks followed by a resting-state recording as outlined in Shader et al. (2021). Participants were seated in a comfortable chair within a sound-attenuated booth facing a monitor screen. A loudspeaker was positioned 1 m from the participant for the free-field presentation of auditory stimuli at 55 dBA.

**Fig. 1:**
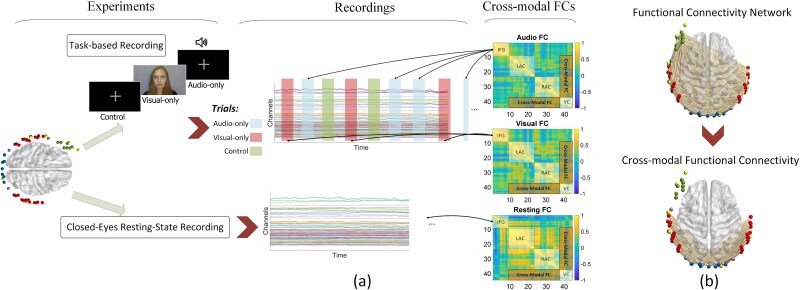
a) Experimental design overview: The experiment comprises two steps. The first step is task-based, involving participants listening to a short story with audio-only, visual speech-only, and control trials. The second step involves a 5-min closed-eye resting state. Based on the recordings from these two steps, adjacency matrices (FC matrices) are calculated for both resting state and task trials. The resting-state FC serves as the baseline to measure task-related components in FC. b) Cross-modal functional connectivity links: illustration of the cross-modal functional connectivity links investigated in this study, depicting connections between auditory and visual cortices.

The experiment was comprised of a task-based component followed by a resting-state component. During the task-based component (Fig. 1a), participants attended to 36 contextually connected segments of a children’s story (“Mrs Tittlemouse” by Beatrix Potter) presented by a female narrator. The story segments were either audio-only or visual-only and had an average length of 12.5 s. In audio-only trials, participants heard the story through the speaker with a fixation gray cross on the screen. During visual-only speech trials, the participants watched a video of a female narrator telling the story without sound. Furthermore, during 10 control segments, the participants attended to a gray fixation cross on the screen with no sound. All segments were followed by 15- to 30-s intervals during which the participants attended to a gray fixation cross on the screen with no sound. Segments were pseudo-randomized in condition while keeping the segments contextually continuous. After the task-based component, we conducted a 5-min closed-eyes resting-state recording. Participants were instructed to close their eyes and relax during the recording.

### fNIRS recording

**Fig. 2:**
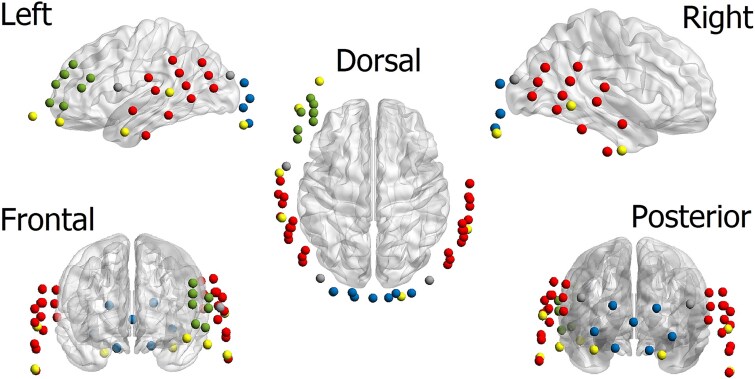
Data acquisition montage: The montage comprises 44 long channels with approximately 3 cm distance between sources and detectors and 8 short channels with an 8 mm separation between sources and detectors. The montage covers the left inferior frontal gyrus (green channels), left and right auditory cortices (red channels), and the occipital lobe (blue channels). Short channels are highlighted in yellow.

Two fNIRS recordings were conducted in this study: one within the first month after implant switch-on and the second at 1 year after implant switch-on. We employed the same fNIRS acquisition setup for both recordings. A continuous-wave NIRScout device (NIRScout, NIRX medical technologies, LLC) was used for data acquisition. The montage comprised 16 sources and 16 photodiode detectors. The sources included two near-infrared light emitters illuminating at wavelengths of ~760 and 850 nm. This configuration established 44 long-channels with an average separation of 3 cm between sources and detectors. The channel placements, as depicted in Fig. 2, covered the inferior frontal gyrus, right and left auditory cortices, and occipital lobe cortical areas of the brain. Additionally, three channels were positioned between these areas, indicated in gray in the figure. The montage also featured 8 short channels with an ~8 mm separation between their sources and detectors.

### Data preprocessing

The MATLAB software, along with the near-infrared spectroscopy (NIRS) toolbox (Santosa et al. 2018), was employed for data preprocessing. Initially, fNIRS recordings were converted to optical density. Channel quality was assessed based on the scalp coupling index (SCI) (Pollonini et al. 2014), with a threshold of 0.5 defining bad channels. Channels with SCI values below 0.5 were excluded from subsequent analysis. The temporal derivative distribution repair method was then applied to the remaining channels to enhance signal quality by mitigating motion artifacts (Fishburn et al. 2019). Subsequently, the optical signals were converted to oxy- and de-oxyhemoglobin (HbO and HbR) using the modified Beer–Lambert Law (Kocsis et al. 2006). Given that long channels capture both cerebral and systemic components and artifacts such as heartbeats, respiration, and Mayer waves, two steps of short-channel correction and band-pass filtering were implemented to mitigate the impact of these artifacts. Short-channel correction involved regressing out short channels (containing systemic artifacts and no cerebral component) from long channels (Santosa et al. 2020). An infinite impulse response (IIR) band-pass Butterworth filter with an order of 7 and a band-pass range of 0.033 to 0.40 Hz was applied to remove low-frequency artifacts like baseline drifts and high-frequency artifacts like heartbeats. Additionally, to address serial correlations in fNIRS time series and reduce spurious correlations, prewhitening was applied before calculating Pearson correlation, as recommended by Santosa et al. (2017).

### Cross-modal functional connectivity

**Fig. 3:**
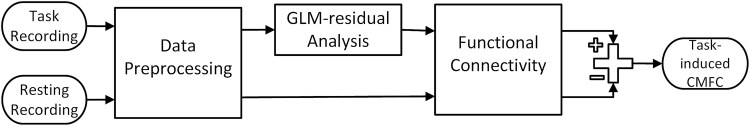
Block diagram representation of the steps applied on the acquired signals to calculate task-related FC.

Following preprocessing, a general linear model (GLM) analysis was applied to task-based recordings to mitigate transient task effects, using canonical HRF-convolved task regressors, akin to the PPI method (Penny et al. 2011; O’Reilly et al. 2012). FC adjacency matrices were calculated for audio-only and visual-only trials based on the residual signals after GLM analysis, as well as for resting-state data. FC was computed using Pearson correlation, with Fisher transformation applied to improve the normality of weight distributions (Fisher 1915). Task-based FC was assessed during 27.5-s intervals of task trials (Fig. 1a) and normalized by subtracting resting-state FC. To reduce spurious components due to windowing, the lower cut-off frequency of the band-pass filter during the preprocessing step was set to approximately 0.036 Hz, corresponding to the inverse of the window length (1/27.5 s; Leonardi and Van De Ville 2015; Zalesky and Breakspear 2015). The study specifically focuses on cross-modal FC links, defined as links connecting nodes in the auditory cortex to nodes in the visual cortex (Fig. 1b). Figure 3 shows a block diagram representing the steps applied on the acquired signals to calculate task-induced FC.

## Results

One of the key objectives of this study was to assess cross-modal plasticity in the primary and secondary auditory cortices concerning task-induced CMFC. To achieve this, we adopted the auditory areas classification in our montage proposed by Shader et al. (2021). Shader et al. employed the fNIRS Optode Location Decider (fOLD) tool to partition the auditory region into two subregions: superior temporal gyrus (STG) and angular gyrus (AG) (They named these areas Herschel’s gyrus and planum temporale in their study), representing distinct anatomical structures of the primary and secondary auditory areas, respectively.

### Task-induced CMFC and CI outcomes: broad auditory areas

Given that our participants received auditory input only through the implanted side, we examined the correlation between average contra- and ipsilateral CMFC links (relative to the implant side) and behavioral speech performance. To test the primary hypothesis, we began by analyzing all channels within the auditory cortex (broad auditory areas) before proceeding to exploratory analyses focused on restricted auditory regions associated with primary and secondary auditory areas. FDR correction was applied separately for each step to account for the exploratory nature of the second phase of analysis.

**Fig. 4:**
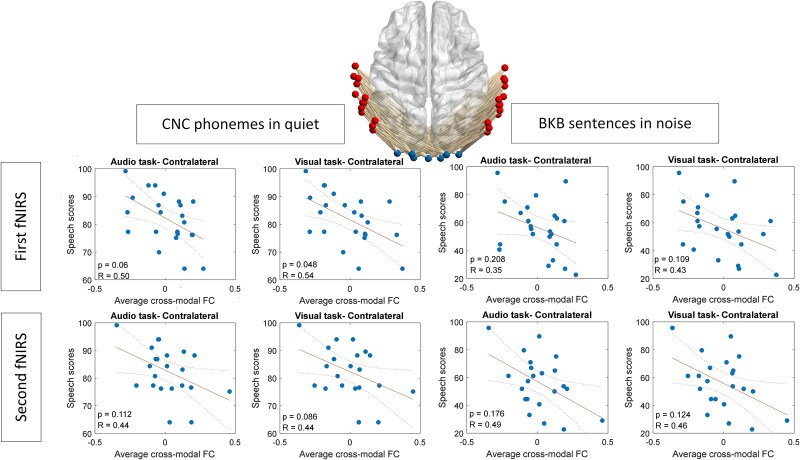
Correlation between average contra-lateral cross-modal functional connectivity (FC) and speech outcomes, employing all channels in the auditory cortex (AC) representing the broad auditory area.

For broad auditory areas, significant or marginally significant negative correlations were observed between average contralateral task-related CMFC during the first fNIRS recording (1 month postimplantation) and CNC phoneme scores in quiet for both visual-only and audio tasks (Fig. 4). All *P*-values were corrected using the Benjamini–Hochberg method. Correlation results are presented exclusively for contralateral links, as analyses showed no significant correlations for ipsilateral links.

### Task-induced CMFC and CI outcomes: restricted auditory areas

Building on the correlations observed in the broad auditory areas, we conducted an exploratory analysis to investigate the origins of contralateral task-related CMFC correlations with speech outcomes. For this purpose, we focused on restricted auditory regions: the AG and the STG. This step aimed to uncover potential differences between primary and secondary auditory areas in their contributions to cross-modal functional connectivity.

**Fig. 5:**
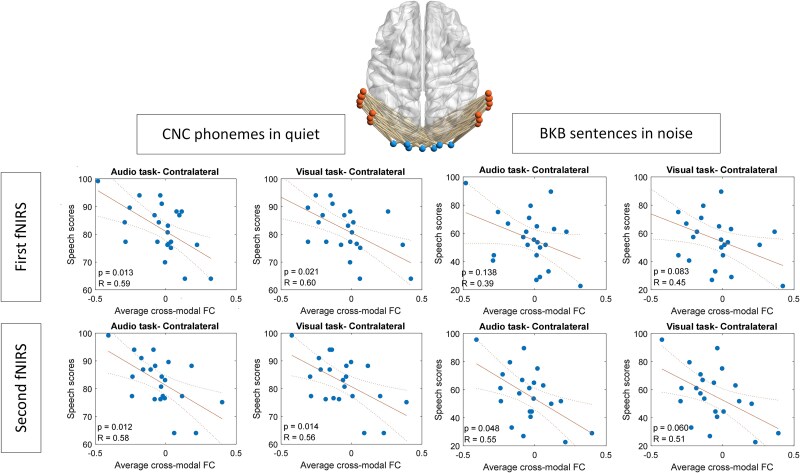
Correlation between average contra-lateral task-induced CMFC and speech outcomes, utilizing FC links from channels in AG (indicated in pink color) to channels in the VC (in blue color).

**Fig. 6:**
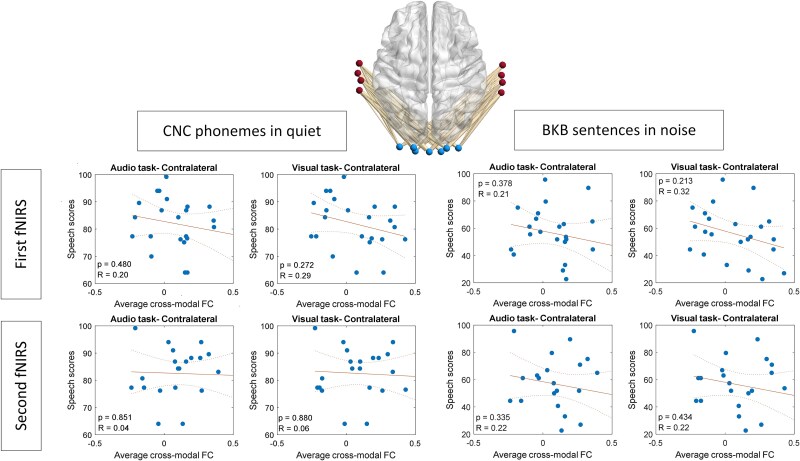
Correlation between average contra-lateral task-induced CMFC and speech outcomes, utilizing FC links from channels in STG (indicated in red color) to channels in the VC (in blue color).

As illustrated in Figs 5 and 6, significant and predominantly negative correlations were observed between speech scores and task-induced CMFC for contralateral links originating from AG to the VC. These findings suggest AG’s stronger involvement in cross-modal plasticity. In contrast, correlations for links originating from STG to VC were weaker and less significant. All *P*-values were adjusted using the Benjamini–Hochberg correction method.

We assessed the potential influence of covariates, including age, deafness duration, and hearing threshold in the nonimplanted ear (PTA), for all cases where task-induced CMFC was significantly correlated with speech scores. Our results indicate that CMFC remains significantly correlated with speech scores even after adjusting for these factors, demonstrating that its association with speech performance is robust and independent of these variables. Furthermore, individual analyses showed that none of the covariates (age, deafness duration, PTA) were significantly correlated with speech scores in our dataset.

### Cross-modal link importance based on CI outcomes

**Fig. 7:**
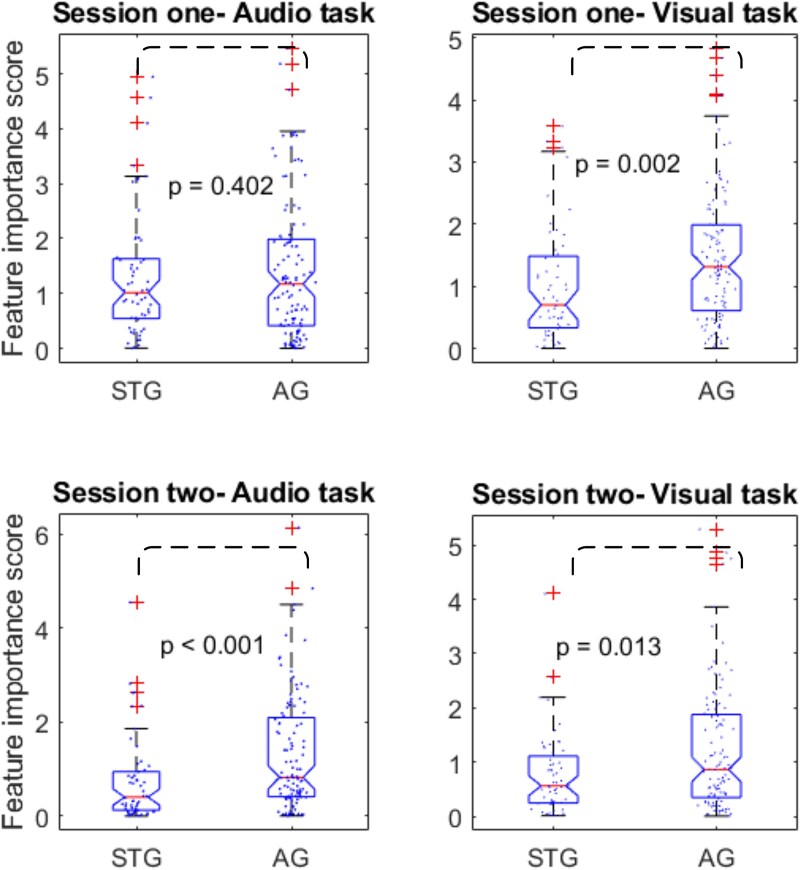
Significance of individual links in predicting performance outcomes in STG and AG. The relative importance was determined using univariate feature ranking for regression via *F*-tests. Links from AG to VC show a stronger correlation with the behavioral speech performance of CI users compared to those from STG. Dashed lines represent two group comparisons.

To better understand the distinction between cross-modal links in AG and STG concerning their correlation with speech performance outcomes one year after CI activation, we employed univariate feature ranking for regression (Waugh 1995). This analysis enabled us to determine the relative significance of individual links in predicting performance outcomes. Using *F*-tests, we assessed the importance of each link in estimating speech performance outcomes, with the negative logarithm of the *P*-values serving as the scores (implemented using the fsrftest function in MATLAB). Consistent with our findings based on average link weights in different restricted areas, we compared significance scores for groups of links from the AG and STG to the VC using unpaired two-sample *t*-tests. The results highlight the higher significance of links from AG in both fNIRS recordings regarding their correlation with speech understanding performance outcomes (Fig. 7).

### Comparing task-induced CMFC of STG and AG after CI

**Fig. 8:**
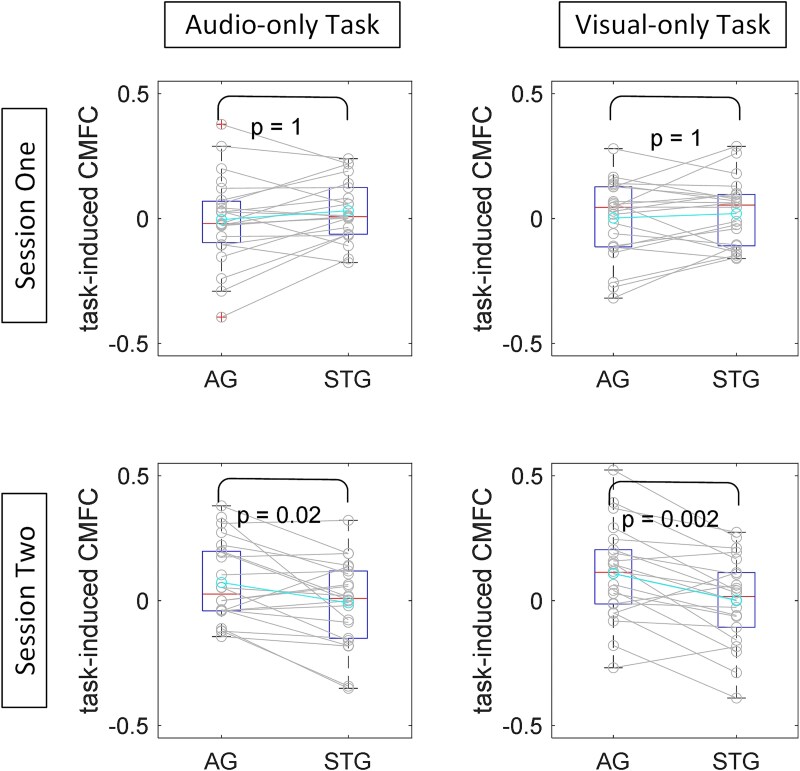
Comparison of average task-induced CMFC between AG and STG links to VC in CI recipients. Initially insignificant shortly after implantation (1 month postdevice switch-on), but after 1 year, task-induced CMFC becomes significantly higher for AG links. Solid lines represent paired *t*-tests. *P*-values are FDR-corrected.

In addition to assessing the correlation between links from the AG and STG and behavioral speech performance outcomes, we compared these weights for the two fNIRS recording sessions conducted 1 month and 1 year postswitch-on. As illustrated in Fig. 8, paired *t*-tests were used to compare the average cross-modal link weights from AG and STG to VC for CI recipients across both recording sessions and various tasks. The results show that, while the difference between average CMFC links was not significant 1 month postimplant switch-on, it became significantly different after 1 year, indicating a higher task-induced CMFC for the AG area.

### Task-induced CMFC and channel pair distances

**Fig. 9:**
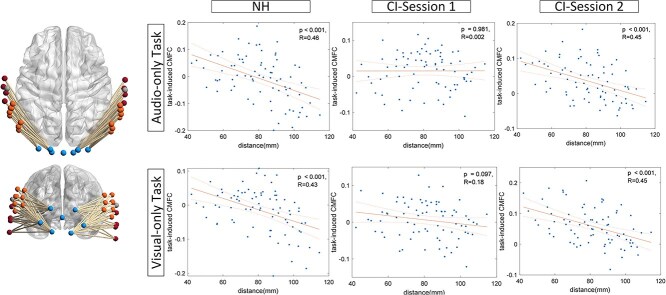
Correlation between task-induced CMFC and the distance between channel pairs within each hemisphere. Notably, for NH subjects, the correlation is negative and significant. Moreover, in the CI user group, the negative correlation strengthens after 1 year of device activation.

Given the notable differences observed in task-induced CMFC of links connecting AG and STG to VC in the results discussed so far, we sought to investigate the relationship between link length and task-induced CMFC. The rationale behind this exploration was the apparent difference between AG and STG in terms of their distance to VC (STG channels being farther away from VC compared to channels in AG). Therefore, we aimed to determine the impact of link length on the task-induced CMFC. In this section, we focused on those links that connect channels in each hemisphere but not across hemispheres. We conducted the same analysis for both NH subjects and CI recipients. As illustrated in Fig. 9, the average changes in task-induced CMFC across subjects negatively correlate with channel pair distance in the NH population. For CI users, our results indicate a weaker correlation shortly after implantation (1 month after CI switch-on), while, after 1 year, a similarly strong negative correlation to the NH group emerges between task-induced CMFC and distance. This implies that topologically closer areas cooperate more (show more correlated activities) to accomplish the audio- and visual-only tasks.

To address potential spatial correlation due to overlapping light paths, we verified that the shortest link length in our setup is 40 mm, which exceeds the spatial range of overlapping paths. Therefore, the observed correlation reflects meaningful functional connectivity rather than an artifact of overlapping signals.

### Task-induced CMFC of CI users compared to NH subjects

**Fig. 10:**
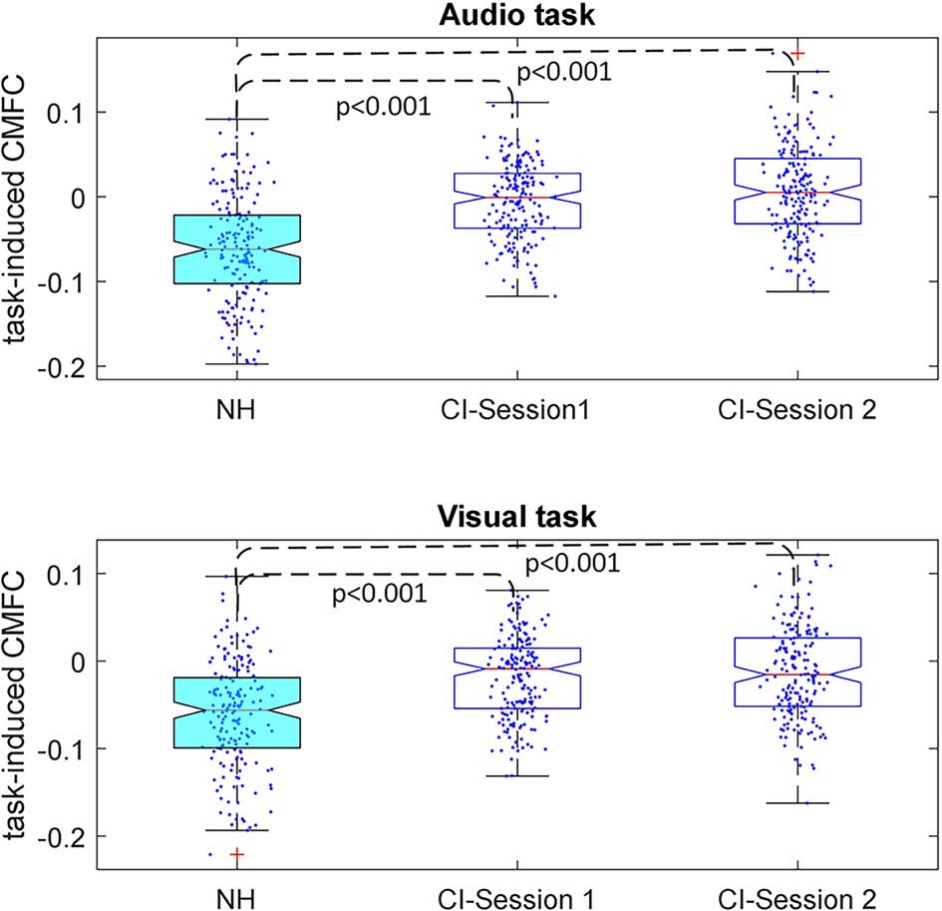
Comparison of the distribution of average task-induced CMFC across subjects for NH and CI groups. CI users exhibit significantly higher task-related CMFC values compared to the NH group (*P*-values represent the significance of the two-group comparison in each recording session). Dashed lines indicate two-group comparisons.

We further investigated the task-induced CMFC of NH subjects and CI users. Figure 10 illustrates the distribution of average link weights across subjects. The results unveil a significantly higher mean value for task-induced CMFC in the CI group compared to NH participants at both time points—1 month and 1 year after device activation.

## Discussion

Cross-modal plasticity underscores the brain’s remarkable capacity for reorganization in response to sensory input. However, several studies have highlighted the constraints and limitations of such reorganization, emphasizing the reliance on preexisting structural circuitry and top–down regulatory mechanisms (Lomber et al. 2010; Meredith et al. 2011; Benetti et al. 2021). This evidence aligns with the notion that the substrate of cross-modal plasticity primarily involves synaptic and functional adaptations, operating at the level of synaptic gain and inhibition, which subsequently lead to changes in functional connectivity without significant large-scale rewiring in the brain (Kral and Sharma 2023). In line with this understanding, our study aimed to assess task-induced changes in CMFC between auditory and visual cortices in postlingually deaf CI recipients, as a critical indicator of cross-modal plasticity following CI activation.

Functional brain imaging was conducted using fNIRS, a noninvasive optical imaging technique that measures changes in oxy- and deoxy-hemoglobin concentration within cortical blood flow. While fNIRS have lower spatial resolution compared to fMRI, its advantages including minimal noise, cost-effectiveness, and compatibility with implant devices make it particularly well suited for hearing research (Chen et al. 2020; Shader et al. 2021).

### Task-induced CMFC measurement methodology

Drawing inspiration from Friston et al.’s PPI method (Friston et al. 1997), we developed an approach to quantify task-induced CMFC (Fig. 3) while addressing two significant limitations inherent in standard PPI applications. Our method incorporates closed-eyes resting-state recordings as a robust baseline, which provides several key advantages.

Firstly, the generalized PPI method, typically used for event-related experiments with multiple randomized tasks, is limited in its ability to isolate the specific effects of individual tasks on FC. Instead, it contrasts the overall effects of tasks relative to a shared baseline (McLaren et al. 2012; Di et al. 2021). This limitation is especially problematic when assessing task-specific changes in CMFC, as required in our study. By utilizing closed-eyes resting-state recordings, we circumvent this limitation and ensure that the individual effects of audio-only and visual-only tasks on FC can be assessed separately.

Secondly, evidence indicates that short control intervals interspersed between task trials are prone to contamination by residual task-related activity, as the brain’s FC may not return to true baseline levels within these intervals (Esmaelpoor et al. 2023). This is further compounded in our experimental design, where participants maintained open eyes during control trials, likely introducing transient visual stimulation (eg fixation cross) or wandering focus. Such residual activity would compromise the reliability of baseline measurements if control intervals were used. By employing closed-eyes resting-state recordings, we ensured that baseline FC measurements were independent of transient task-related activity and visual input. This approach effectively resolved the limitations associated with short control intervals and enabled a more precise assessment of task-induced CMFC changes. Moreover, the closed-eyes baseline provided a consistent and controlled reference point, enhancing the reliability and accuracy of our results, particularly when analyzing cross-modal plasticity in response to visual-speech tasks.

This methodological refinement not only addresses key challenges in using PPI for CMFC studies but also ensures a more robust foundation for interpreting task-induced connectivity changes in the context of cross-modal plasticity.

### Distinct patterns of cross-modal plasticity revealed in auditory cortex subregions

In our investigation of the correlation between task-related CMFC and speech performance outcomes among CI users, we initially focused on the broad areas within the auditory cortex encompassed by our montage setup. Our only precondition was the classification of cross-modal links into contra and ipsilateral categories relative to the implant side, reflecting the experimental conditions where participants received auditory stimuli through the cochlear implant on one side. Given the well-established lateralization patterns in structural auditory pathways (Pickles 2015), this distinction was deemed crucial. Our findings unveiled a notable negative correlation, with some instances reaching statistical significance (at α = 0.05), between average contra-lateral task-related CMFC observed in fNIRS recordings obtained in 1 month postdevice activation and those taken after 1 year, and the speech understanding scores recorded after 1 year (Fig. 4).

Building on these findings, we conducted exploratory analyses focusing on restricted subregions within the auditory cortex, informed by prior research (Shader et al. 2021). This analysis revealed significant differences between AG and STG concerning their correlation with the behavioral speech outcomes of the links originating from channels within these regions to VC. Specifically, our results indicated that the observed correlation, predominantly evident in the initial examination of broad areas, was largely driven by links originating from AG to VC (Figs. 5 and 6). To confirm these disparities, we ranked all links and found notably higher significance scores for links originating from AG in terms of their correlation with speech scores (Fig. 7). Furthermore, our assessment of brain plasticity revealed a significant difference emerging after 1 year in task-related CMFC for links from AG to VC compared to those from STG to VC, with higher amplitude observed for AG links (Fig. 8).

These findings underscore a distinct divergence between AG and STG concerning cross-modal plasticity postcochlear implantation, as assessed through task-induced CMFC. They emphasize the heightened significance of the AG area in facilitating cross-modal cooperation with VC, alongside the more pronounced plastic changes evidenced by significantly higher amplitude CMFC of links originating from this area to VC compared to links from STG to VC 1 year post switch-on. Thus, our results reaffirm studies indicating cross-modal activities after hearing loss predominantly relate to specific auditory regions, primarily associated with secondary auditory processing regions (Battal et al. 2019; Benetti et al. 2021; Kral and Sharma 2023).

### Link length and task-induced CMFC: insights into brain economy

One notable topological difference between AG and STG lies in their respective distances from VC. This prompted us to investigate whether link length, defined as the distance between pairs of channels, could influence task-induced CMFC. In essence, we sought to determine whether closer areas in AC and VC exhibit greater cooperation in task performance. Given the brain’s economic considerations, it’s reasonable to expect that shorter links would experience more pronounced task-related changes in FC. Neural elements and their connections are constrained by limited brain volume, making material and metabolic costs a significant concern. However, brain networks must also prioritize topological efficiency, robustness, and computational performance. Thus, the placement of neuronal components should ideally be optimized to balance these competing demands (Bullmore and Sporns 2012).

To explore this further, we examined the correlation between link length and task-related FC for links within each hemisphere in both CI users and NH subjects. As the physical connections between nodes in VC and AC located in different hemispheres are not straightforward and Euclidean distance may not be a proper estimation, we focused on links within each hemisphere. Our results (Fig. 9) demonstrated that, overall, in the NH group, task-related FC tends to decrease as the distance between nodes increases, showing a highly significant negative correlation. Shortly after implantation, CI users exhibited a weaker negative correlation, but after one year, a similar trend emerged. This suggests that the brain requires time to adapt to the new stimulation provided by the implant device, as indicated by user performance, where most changes occur within the first 6 months and stabilize after 1 year. These findings, in line with the brain’s economy principle, imply that plastic changes related to CMFC after implantation primarily adhere to principles of neural efficiency, showing higher task-related CMFC correlation for links between closer channels (Bullmore and Sporns 2012).

### Enhanced task-induced CMFC in cochlear implant users

Upon comparison of task-induced CMFC during both visual-speech and audio-only tasks between NH individuals and CI users, a significant distinction emerged: CI users displayed a notably higher mean task-induced CMFC compared to the NH group (Fig. 10). This finding indicates a stronger correlation in cross-modal brain activities during tasks among CI users than among NH subjects. Essentially, CI users, similar to individuals with hearing loss, exhibit an amplified reliance on cross-modal activities to comprehend both audio and visual speech. This dependence might be attributed to the cochlear implant’s delivery of signals with coarse spectral content in contrast to the natural acoustic hearing experienced by NH individuals, coupled with structural changes in the hearing system following hearing loss (Fallon et al. 2008; Wilson and Dorman 2008), which requires more pronounced CI users’ reliance on cross-modal activities even after 1 year of using the device.

## Conclusion and future directions

This study explored cross-modal plasticity among CI recipients with severe hearing loss in the contralateral ear, focusing on task-induced CMFC. To do so, we proposed a robust method inspired by PPI while addressing its limitations. We found a negative correlation between average contralateral task-induced CMFC and speech outcomes, particularly pronounced in links originating from AG, both 1 month and 1 year postdevice activation. Further distinctions between primary and secondary auditory areas, specifically AG and STG, revealed plastic cross-modal changes in 1 year postswitch-on, resulting in higher task-induced CMFC in AG areas compared to STG, aligning with principles of neural efficiency. Task-induced CMFC remained elevated in CI users compared to NH cohorts even after 1 year postimplantation, suggesting a sustained reliance on cross-modal activities. Future studies could extend these findings by exploring more prolonged time intervals beyond 1 year to better understand long-term plasticity. Additionally, investigating a more comprehensive model of connectivity links, rather than restricting analyses to specific regions as in this study, could provide deeper insights into cross-modal plasticity and its implications for CI outcomes.
